# The Optimization of Viscozyme-Assisted Hydrolysis to Enhance Protein Extraction from VD20 Broken Rice: A Response Surface Approach for Functional Health Applications

**DOI:** 10.3390/foods15071265

**Published:** 2026-04-07

**Authors:** Do Trang Minh Pham, Trung Tinh Vo, Xuan Phong Huynh, Hanh Uyen Le, Binh An Pham, Chi Khang Van, Long Giang Bach

**Affiliations:** 1 Institute of Food and Biotechnology, Can Tho University, Can Tho City 900000, Vietnam; pdtrangminh@tgu.edu.vn (D.T.M.P.); hxphong@ctu.edu.vn (X.P.H.); 2 Faculty of Agriculture and Food Technology, Tien Giang University, Dong Thap Province 870000, Vietnam; 3 Institute of Applied Technology and Sustainable Development, Nguyen Tat Thanh University, Ho Chi Minh City 700000, Vietnam; vttinh@ntt.edu.vn (T.T.V.); lhuyen@ntt.edu.vn (H.U.L.); pban@ntt.edu.vn (B.A.P.); vckhang@ntt.edu.vn (C.K.V.); 4 Center for Hi-Tech Development, Nguyen Tat Thanh University, Saigon Hi-Tech Park, Ho Chi Minh City 700000, Vietnam

**Keywords:** VD20 broken rice, Viscozyme, Box–Behnken, flour, hydrolysis

## Abstract

This study investigated and optimized the cellulose hydrolysis conditions of VD20 broken rice flour using Viscozyme to enhance protein content and protein recovery efficiency. Four processing variables, including pH (5.0–6.0), temperature (40–60 °C), enzyme concentration (20–40 U/g), and hydrolysis time (34–38 h), were simultaneously evaluated using response surface methodology. The results indicated that temperature, enzyme concentration, and hydrolysis time influenced protein release and recovery, whereas pH had a limited effect within the studied range. All variables exhibited nonlinear effects, with distinct optimal regions beyond which protein extraction efficiency declined. The maximum protein content of approximately 77% was obtained at a pH of 5.56, temperature of about 55 °C, enzyme concentration of 30 U/g, and hydrolysis time of 36 h. In contrast, the highest protein recovery efficiency of approximately 54–55% was achieved at a pH of 5.53–5.57, temperature of about 53 °C, enzyme concentration of about 30 U/g, and hydrolysis time of about 36 h. Simultaneous optimization of both responses identified balanced conditions at a pH of about 5.53, temperature of about 53 °C, enzyme concentration of about 29 U/g, and hydrolysis time of about 36 h, yielding protein content of approximately 76–77% and protein recovery efficiency of about 55%. These findings demonstrate that optimization provides an effective strategy for maximizing protein utilization from VD20 broken rice and support the application of Viscozyme-assisted hydrolysis in the valorization of rice processing by-products.

## 1. Introduction

Broken rice, particularly from the VD20 rice variety, is a plentiful agro-industrial by-product of the rice milling process. The VD20 variety is a fragrant, short-duration cultivar widely grown in Vietnam’s Mekong Delta and yields significant quantities of broken kernels during milling [1]. Importantly, broken rice retains a nutritional composition equivalent to that of whole rice grains, including its carbohydrate-rich endosperm and protein content. Prior studies have indicated that broken rice contains roughly 6–8% protein by weight (dry basis), which is similar to intact milled rice [2]. Despite this appreciable protein content, broken rice is often undervalued, being sold cheaply or used in low-grade applications. Therefore, utilizing this resource for protein extraction not only adds economic value but also aligns with the sustainable use of agricultural by-products. Indeed, recent studies on VD20 broken rice have demonstrated its potential in value-added products and highlighted the significance of its protein and micronutrient profile [1]. These findings provide essential platforms for exploring efficient protein recovery from VD20 broken rice flour.

Rice protein has gained attention in both food and feed industries due to its favorable amino acid profile, functionality, and hypoallergenic nature. Rice grain proteins comprise all 17 common amino acids, including nine essential amino acids, in a well-balanced proportion [3]. Rice protein is relatively lysine-rich for cereal, contributing to its high digestibility (around 90–93%) and biological value (~74%) [4]. This excellent digestibility and amino acid composition make rice protein nutritionally competitive with animal proteins and superior to many other cereal proteins. Moreover, rice protein is naturally gluten-free and exhibits minimal allergenicity, which is advantageous for sensitive consumers. In terms of functionality, rice proteins (primarily glutelin and albumin fractions) can provide useful technofunctional properties. However, native rice proteins tend to have low water solubility at near-neutral pH and an intact globular structure, which can limit their direct use in formulations. Therefore, improving the solubility and extractability of rice protein is crucial to maximizing its bioavailability and functionality. Given its high nutritional quality and generally bland flavor, rice protein is increasingly sought as a plant-based protein ingredient in foods, beverages, and animal feeds [5]. Optimizing extraction from abundant sources like broken rice could further expand its applications in fortified foods, feed supplements, and specialized diets.

Multiple methods exist to extract protein from rice and its by-products with distinct advantages. Traditional chemical extraction (e.g., alkaline solubilization followed by acid precipitation) is widely used for rice protein isolates due to its high efficiency. More than 80% of rice proteins (particularly from broken rice) are soluble in alkali, and alkaline extraction can yield a high protein recovery [3]. However, harsh alkaline conditions (e.g., strong NaOH at high pH) may partially degrade essential amino acids and denature proteins, potentially reducing nutritional and functional qualities. For example, excessive alkalinity can promote Maillard reactions or lysine loss in rice protein isolates. In contrast, enzymatic extraction has emerged as a milder and more specific approach to isolating proteins from plant materials. Enzyme-assisted methods use carbohydrases or proteases to break down cell wall polysaccharides and release protein without extreme pH or temperature, thus preserving protein integrity [6]. In the context of rice and cereal by-products, cellulase-rich enzyme cocktails have shown particular promise. Viscozyme, which is a multi-enzyme complex containing cellulases, hemicellulases, β-glucanase, xylanase, and pectinase, can hydrolyze structural carbohydrates in the rice matrix. By degrading the cellulose and hemicellulose network of rice bran or endosperm cell walls. Viscozyme treatment facilitates the release of proteins that would otherwise remain bound within the insoluble matrix. Viscozyme was selected due to its synergistic multi-enzyme activities that effectively degrade plant cell walls, thereby enhancing intracellular protein release and extraction efficiency. This enzymatic strategy operates under relatively mild conditions (typically pH 4–6, 40–55 °C), avoiding the protein denaturation associated with strong chemicals. Recent research has demonstrated the efficacy of Viscozyme-assisted extraction in enhancing protein yield and functionality. For instance, applying Viscozyme pretreatment to soy okara (a protein-rich fiber by-product) at optimal conditions (53 °C, pH 6.2) increased the protein content in the extract by 17% and protein recovery by 86% relative to no enzyme pretreatment [7]. Similarly, Rodrigues et al. (2014) reported that Viscozyme and related carbohydrases improved protein extractability from rice bran under neutral pH conditions [6]. These studies highlight the advantages of enzyme-assisted extraction, including enhanced protein yields under mild processing conditions and the production of protein concentrates that retain intact amino acid profiles. Therefore, using Viscozyme to extract protein from VD20 broken rice flour is a promising approach, but it requires careful optimization to maximize efficiency.

Despite the known potential of enzymatic extraction, there is a notable gap in systematic research on optimizing these conditions for broken rice. Most prior studies on rice protein extraction have focused on chemical methods or examined other substrates (e.g., rice bran and okara) rather than broken milled rice. Although enzymatic extraction methods provide milder conditions and higher specificity, they often suffer from high enzyme cost, long processing times, and incomplete protein recovery efficiency. Despite these advances, existing extraction techniques remain suboptimal, highlighting the need for systematic optimization to improve both protein yield and quality. To date, very few reports have addressed enzyme-assisted protein extraction from broken rice grains and specifically optimized the process for the VD20 rice variety. The complex interplay of several factors, such as enzyme concentration, incubation time, temperature, and pH, can influence the protein efficiency and quality of broken rice. Without optimization, suboptimal conditions might lead to incomplete hydrolysis of the rice matrix or unnecessary enzyme usage, resulting in lower recovery or higher costs. Therefore, a systematic approach is required to identify the ideal combination of processing parameters for maximum protein extraction. Response surface methodology (RSM) offers a powerful statistical tool for this purpose. In particular, Box–Behnken design enables the evaluation of multiple factors and their interactions with a limited number of experimental runs. RSM has been successfully applied in optimizing protein extraction processes in similar contexts. Recent studies have highlighted the effectiveness of enzymatic extraction of rice protein, particularly when using multi-enzyme systems or optimized conditions. For example, de Figueiredo et al. (2018) used a rotatable central composite design to optimize Viscozyme-assisted extraction from okara, achieving clear optima for both protein content and recovery [7]. Bandyopadhyay et al. (2012) reported that Viscozyme-assisted hydrolysis of defatted rice bran achieved a high protein recovery of approximately 82.6% due to efficient degradation of cell wall components [8]. Similarly, Leal et al. (2021) also demonstrated that cellulase-based extraction under optimized conditions yielded protein recovery of about 63–72%, depending on processing parameters [9]. By employing an RSM approach (Box–Behnken design), the present study investigated how Viscozyme hydrolysis conditions affected protein content and recovery efficiency from VD20 broken rice. This approach not only identified optimum conditions statistically but also provided insights into the relative influence of each factor and any synergistic interactions, thereby contributing novel knowledge regarding the valorization of broken rice protein and providing guidance for scalable protein recovery in the food and feed industries. The novelty of this work lies in the multi-objective optimization approach, which balances both yield and quality to improve protein utilization from rice processing by-products.

## 2. Materials and Methods

### 2.1. Materials and Chemicals

The rice variety used in this study was VD20, which was harvested and purchased from Go Cong Tay District, Dong Thap Province, Vietnam. Broken rice was ground using a dry milling process with a laboratory grinder (Seka, Ha Noi City, Vietnam) and subsequently sieved to obtain a uniform particle size of less than 250 µm. Viscozyme, produced from *Aspergillus* sp., was used as the enzyme source. This enzyme is capable of hydrolyzing polysaccharides, including cellulose, beta-glucan, hemicellulose, and xylan, with a declared activity of 1000 U/g (Sigma, Ronkonkoma, NY, USA). One unit (U) of enzyme activity is defined as the amount of enzyme required to catalyze the conversion of 1 µmol of substrate per minute under specified conditions (pH, temperature, and substrate). The activity, expressed as U/g, corresponds to the total enzymatic activity per gram of enzyme preparation. According to the manufacturer, the optimal pH and temperature ranges of the enzyme are 3.0–6.5 and 50–70 °C, respectively. Prior to use, enzyme activity was verified experimentally, and the enzyme solution was diluted to obtain the desired activity levels for each experiment.

### 2.2. Protein Extraction Process

Rice flour slurry was gelatinized at 90 °C for 20 min after adjustment of the selected solid concentration and pH. The gelatinized slurry was then liquefied with alpha-amylase at 30 U/g, pH 6.0, and 80 °C for 30 min, followed by enzyme inactivation at boiling temperature for 10 min. The liquefied hydrolysate was subsequently saccharified with gamma-amylase at 30 U/g, pH 5.0, and 60 °C for 80 min, and then the enzyme was inactivated by boiling for 10 min. The hydrolysate obtained after saccharification was used as the starting material for protein extraction with Viscozyme. For each experimental unit, 50 mL of hydrolysate was adjusted to pH 4.0–6.0, and Viscozyme was added at 10–50 U/g. Enzymatic hydrolysis was carried out at 30–60 °C for 18–42 h. After hydrolysis, the enzyme was inactivated by heating at boiling temperature for 10 min, and the samples were cooled to room temperature and filtered to separate the liquid fraction from the residual solids. The resulting samples were then analyzed for protein content and protein recovery efficiency. Preliminary screening experiments were conducted to select appropriate levels of pH, temperature, enzyme concentration, and hydrolysis time for the subsequent Box–Behnken optimization. The Box–Behnken optimization of these factors was presented separately in the subsequent section.

### 2.3. Determination of Protein Content

Protein content was determined using the Kjeldahl method [10]. Solid samples (1 g) or liquid samples (1 mL) were digested with concentrated sulfuric acid in the presence of a catalyst mixture (K_2_SO_4_:CuSO_4_:Se, 100:10:1 ratio) until complete mineralization. The released ammonia was distilled after alkalization with sodium hydroxide, absorbed in boric acid solution, and titrated with 0.1 N sulfuric acid.

Total nitrogen was calculated according to the following equations:

%N = (V × 0.0014/m) × 100

where *V* is the volume of 0.1 N H_2_SO_4_ (mL), *m* is the sample mass (g), and 0.0014 is the nitrogen equivalent (g) per mL of 0.1 N H_2_SO_4_. Protein content was calculated as

%Protein = %N × 6.25

### 2.4. Determination of Protein Recovery Efficiency

Protein recovery efficiency from rice was determined by comparing the amount of protein recovered in the liquid extract after enzymatic treatment to the initial protein present in the rice sample. After hydrolysis, the liquid fraction was separated via filtration, and the protein concentration in the filtrate was measured using the Kjeldahl method to determine the protein content of the extract [11].

$$\mathrm{Protein}\;\mathrm{recovery}\;\mathrm{efficiency}\;(\%)=\mathrm{Cp}\times \frac{V}{m0}\times \frac{P0}{100}\times 100$$

where Cp is the protein concentration in the extract (g/L), V is the volume of the protein extract (L), m_0_ is the mass of the rice sample used for extraction on a dry basis (g), and P_0_ is the initial protein content of the rice sample before extraction (%).

### 2.5. Data Analysis

For the optimization experiments, Portable Statgraphics Centurion 15.2.11.0 software was used to design the experiments according to RSM, employing a Box–Behnken design with four independent factors (X_1_, X_2_, X_3_, and X_4_). Three replicates were performed at the center point to estimate the experimental error. The experimental results were expressed as the mean ± standard deviation, and graphical representations were generated using Microsoft Excel 2019.

Each factor was investigated at three coding levels: −1 (low level), 0 (central level), and +1 (high level). Experiments were arranged at the midpoints of the edges of the multidimensional cube, enabling the simultaneous evaluation of individual effects and interactions between pairs of factors, while avoiding experimental points located at the extreme vertices of the investigation space. With four factors, the total number of experiments was determined by the formula *N* = 2*k*(*k* − 1) + *C*_0_, where *k* is the number of factors investigated, and *C*_0_ is the number of centers. Therefore, with *k* = 4 and three centers, the total number of experiments performed was 27. The experimental runs were conducted in a randomized order to minimize the effects of uncontrolled variables.

## 3. Results and Discussion

### 3.1. Effect of Cellulose Hydrolysis Conditions in VD20 Broken Rice Flour Using Viscozyme on Protein Content

During cellulose hydrolysis using enzymes for protein recovery from rice flour, protein recovery efficiency was affected by processing conditions. Among these, reaction pH (X_1_), temperature (X_2_), enzyme concentration (X_3_), and hydrolysis time (X_4_) were identified as critical factors governing enzyme activity, the extent of starch structure degradation, and the efficiency of protein release. These factors not only exerted individual effects but also interacted with one another, thereby altering protein recovery efficiency. Therefore, simultaneous investigation and optimization of these four factors were necessary to enhance protein recovery efficiency from rice flour during enzymatic cellulose hydrolysis. After the cellulose hydrolysis stage, the experimental results showed suitable values for the investigated factors, including a pH of 5.5, a reaction temperature of 50 °C, an enzyme concentration of 30 U/g, and a hydrolysis time of 36 h. Based on these results, an optimization model comprising four factors and their respective levels was developed. The experimental design for the optimization of the cellulose hydrolysis process was established using the Box–Behnken model, as presented in Table 1. The experimental findings indicated that pH values in the range of 5.0–6.0, temperatures between 40 and 60 °C, enzyme concentrations of 20–40 U/g, and hydrolysis times of 34–38 h had significant effects on the protein extraction process. Consequently, optimization using a four-factor Box–Behnken design was conducted to determine the optimal parameters for cellulose hydrolysis of broken VD20 rice flour in order to achieve the maximum protein content.

The objective of the optimization model was to identify hydrolysis conditions that maximized protein content while maintaining process feasibility. Accordingly, the study proceeded in sequence from preliminary range selection to Box–Behnken design construction, experimental implementation under the designed conditions, and regression analysis for model development and optimization.

Table 2 shows that the first three-order factors, namely, temperature, enzyme concentration, and hydrolysis time, had statistically significant effects on protein content, with corresponding *p*-values of 0.0047, 0.0139, and 0.0146, respectively. Among these factors, temperature exhibited the most significant influence (F = 211.22), highlighting its decisive role in enzyme activation and promotion of the hydrolysis process. The enzyme concentration and reaction time also played important roles, indicating a direct relationship between the amount of catalytic enzyme, the extent of hydrolysis, and the requirement for sufficient reaction time for the system to approach equilibrium.

In contrast, pH showed a *p*-value of 0.0675, which did not reach the significance level of 0.05 within the investigated range, suggesting a limited effect of pH on protein content. This result was consistent with Hadinoto et al. (2024), who reported that pH mainly regulated protein solubility and secondary structure, yet was not a determining factor once enzyme operating conditions had been optimized [12]. These observations were in agreement with the principles of enzyme kinetics and stability: increasing temperature within the optimal activity range enhanced collision frequency and enzyme–substrate complex formation, while enzyme concentration and reaction time governed the extent to which hydrolysis approached equilibrium. Conversely, when pH remained within a narrow activation range, minor variations were insufficient to cause substantial differences in protein recovery efficiency [13].

All quadratic terms (X_1_^2^, X_2_^2^, X_3_^2^, X_4_^2^) exhibited very strong statistical significance (*p* ≤ 0.0014), with exceptionally high F-values ranging from 724.02 to 855.35, reflecting the nonlinear behavior of the system. This indicated that each factor possessed its own optimal point, beyond which protein recovery efficiency declined due to enzyme inactivation, protein denaturation, or changes in substrate permeability. Consequently, the quadratic model provided a more accurate description of the relationship between reaction conditions and the response variable compared with a linear model. This finding was consistent with recent trends in enzymatic protein extraction optimization, where response surfaces typically exhibited distinct optimal peaks and declining regions when individual factors were excessively increased or decreased [14].

Interaction effects among X_1_X_2_, X_1_X_3_, X_2_X_3_, X_2_X_4_, and X_3_X_4_ were all statistically significant (*p* < 0.05). The interaction between pH and temperature (F = 61.69) demonstrated a synergistic effect on maintaining enzyme activity, similar to that reported by Sedlar et al. (2025) for protein extraction from cauliflower leaves and broccoli florets [15]. Specifically, at near-optimal pH values, increasing temperature enhanced catalytic activity, yet when pH deviated substantially from the optimal range, elevated temperature accelerated enzyme deactivation. The interaction between enzyme concentration and reaction time (F = 24.29) indicated that increasing enzyme dosage was effective only when sufficient time was provided to complete hydrolysis. Under short reaction times, excess enzyme contributed little to improving extraction efficiency. Similarly, the interaction between temperature and time (F = 20.87) revealed that prolonged reaction time was beneficial only within an acceptable temperature range; exceeding the optimal temperature led to reduced efficiency due to enzyme degradation. These results were consistent with previous studies on enzymatic protein extraction from plant materials and agricultural by-products, confirming the coordinated roles of pH, temperature and enzyme concentration–time interactions in optimizing reaction efficiency [15].

Regarding model adequacy, the lack-of-fit test yielded a *p*-value of 0.0549 (>0.05), indicating no significant difference between predicted and experimental values, thereby confirming the suitability of the quadratic model. The small pure error (mean square pure error = 0.197433) further demonstrated good experimental reproducibility. The simultaneous presence of significant linear, quadratic, and interaction effects, together with high F-values and *p*-values below 0.05 for most terms, supported the selection of a second-order model for multivariate optimization.

To describe the relationship between cellulose hydrolysis conditions and protein content, a second-order regression equation was established as follows:

(1) Protein (%) = −2393.86 + 267.048X_1_ − 0.984X_2_ + 3.60133X_3_ + 93.7942X_4_ − 22.5083X_1_^2^ + 0.394X_1_X_2_ − 0.269X_1_X_3_ − 0.775X_1_X_4_ − 0.0208333X_2_^2^ − 0.016425X_2_X_3_ + 0.05075X_2_X_4_ − 0.0527333X_3_^2^ + 0.05475X_3_X_4_ − 1.29427X_4_^2^

where X_1_ represents the pH (5.0–6.0);

X_2_ represents the temperature (40–60 °C);

X_3_ represents the cellulase enzyme concentration (20–40 U/g);

X_4_ represents the hydrolysis time (34–38 h).

**Figure 1:**
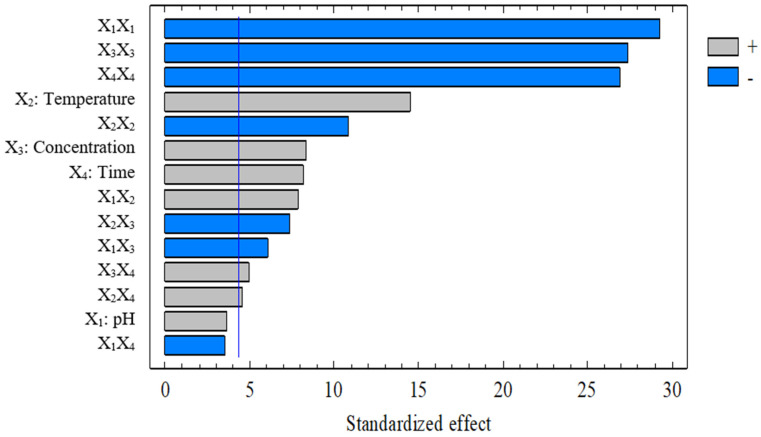
The effect of variables on protein content during cellulose hydrolysis via Viscozyme.

Figure 1 ranks the standardized effects of the investigated factors and their interactions on protein content. Positive and negative bars reflect the direction of the standardized effects, whereas the relative bar length indicates their magnitude. The most prominent contributions were associated with the quadratic terms, particularly those of pH, enzyme concentration, and hydrolysis time, confirming that protein content changed nonlinearly across the investigated domain and that optimal operating regions existed for the process. This interpretation was consistent with the second-order model derived from ANOVA and previous reports on enzyme-assisted protein extraction from plant matrices [16]. Among the linear terms, temperature showed the largest effect, followed by enzyme concentration and hydrolysis time, whereas pH contributed less within the investigated range. This trend was in line with the catalytic characteristics of Viscozyme, for which temperature influences reaction kinetics and disrupts the polysaccharide matrix surrounding the protein [16]. The Pareto plot also showed that interaction effects contributed to the response, particularly the temperature–enzyme concentration combination, whereas the pH–time combination was comparatively weak. Overall, the Pareto analysis demonstrated that processing parameters during the Viscozyme-assisted hydrolysis stage exerted significant and nonlinear effects on the recovered protein content. Simultaneous consideration of linear, quadratic, and interaction effects was therefore essential for constructing an accurate optimization model, contributing to improved protein recovery efficiency from broken rice, a promising yet underutilized agro-industrial by-product.

**Figure 2:**
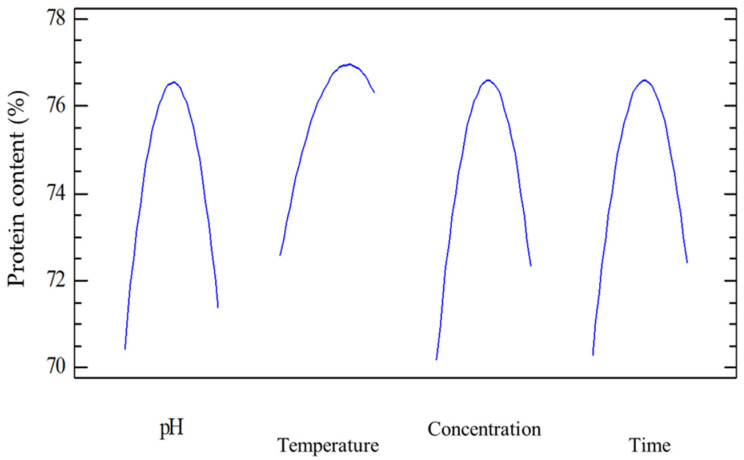
Effects of pH, temperature, concentration, and time on protein content.

Figure 2 illustrates the nonlinear effects of pH, temperature, enzyme concentration, and reaction time on the protein content obtained during enzymatic protein extraction. All four curves exhibited downward-opening parabolic shapes, indicating the presence of optimal operating conditions at which protein content reached a maximum. These trends were consistent with ANOVA and Pareto analyses, in which all quadratic terms were highly statistically significant, thereby confirming the nonlinear nature of the enzymatic response.

Effect of pH: protein content increased as pH approached the optimal range, then decreased markedly when pH exceeded this threshold. Specifically, protein yield increased from acidic to near-neutral conditions, reached a maximum at the optimal pH, and subsequently declined sharply. This behavior reflected the intrinsic catalytic characteristics of enzymes, which have a specific optimal pH that maintains the three-dimensional structure of the active site [17]. Deviations from this optimal pH disrupted the tertiary structure of the enzyme, reduced catalytic activity, and consequently lowered extraction efficiency.

Effect of temperature: The protein content also increased with temperature up to a certain point and then decreased due to enzyme denaturation at temperatures exceeding the optimum. This trend was consistent with the Arrhenius principle, whereby increasing temperature enhanced reaction rates until thermal denaturation of the enzyme occurred [18]. This occurred because higher temperatures increasingly drove the enzyme into inactive conformations (often on timescales faster than thermal denaturation), so that above the optimum temperature, most of the enzyme existed in nonproductive forms [19]. Temperature also altered substrate accessibility. Moderate heating partially unfolded or swelled protein substrates, thereby exposing more peptide bonds to the enzyme [20]. The optimal temperature of microbial-derived protein-hydrolyzing enzymes typically ranges from 50 to 60 °C. These findings were in agreement with Amiri et al. (2024) [21], where optimization of enzymatic reaction temperature was the key determinant of improving protein recovery efficiency during microalgae extraction. This mechanism reflected enzyme–substrate kinetics and the structural stability of enzymes within a limited temperature range [21].

Effect of enzyme concentration: As the enzyme concentration increased, protein content increased rapidly due to the greater availability of catalytic sites, yet eventually reached a saturation point when substrate availability became limiting [22]. Beyond this point, further increases in enzyme concentration did not improve reaction efficiency and lead to inhibitory effects. According to Michaelis–Menten kinetics, when substrate was abundant, the reaction rate increased linearly with the enzyme concentration, so higher enzyme levels drove greater protein release. However, once the enzyme concentration became high enough that substrate availability or product release was limited, adding more enzyme had little effect [23]. This phenomenon has been widely reported in numerous RSM-based studies involving enzymatic protein extraction, where the interaction between the enzyme concentration and reaction time influenced the overall reaction performance [24].

Effect of reaction time: The protein content increased with reaction time and reached a maximum level at a specific duration. Prolonging the reaction beyond this optimal time resulted in a slight decrease in protein content, likely due to excessive hydrolysis that disrupted soluble protein structures. Initially, the protein yield rose as the reaction proceeded, but the curve flattened as the substrate was consumed. Eventually, a plateau was reached when most extractable protein had been released, enzymatic cleavage sites were exhausted, and protein release slowed to near zero [25].

**Figure 3:**
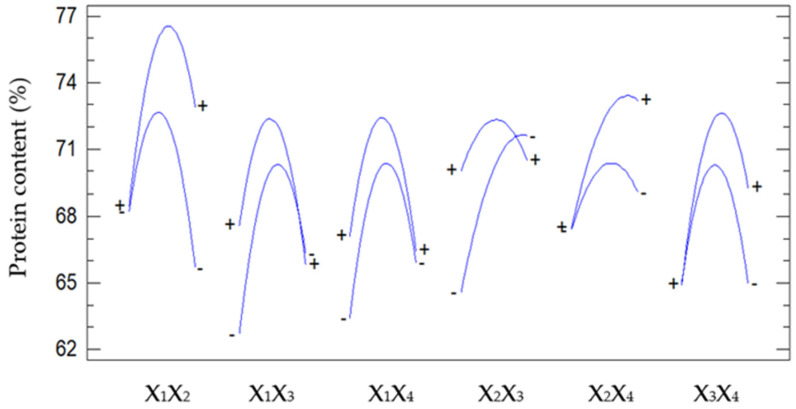
Effect of each pair of factors on protein content.

Figure 3 illustrates the interactive effects of pairs of factors—pH (X_1_), temperature (X_2_), enzyme concentration (X_3_), and hydrolysis time (X_4_)—on protein content. The response surfaces revealed nonlinear variations in protein content as a function of each factor pair and demonstrated differences in the magnitude of influence among the interactions. Among the investigated interactions, the interaction between pH and temperature (X_1_X_2_) exhibited the largest amplitude of variation in protein content, with a maximum observed in the slightly acidic to near-neutral pH range (approximately 5.5–6.0) and at a temperature close to 50 °C. This finding indicates that the activity of the employed enzymatic system, particularly Viscozyme, was dependent on the combined effects of these two parameters.

The interaction between pH and enzyme concentration (X_1_X_3_) also exerted a significant effect on protein content. When pH remained within the optimal range, increasing the enzyme concentration resulted in a significant increase in protein content. However, at pH values below or above the optimal range, the effectiveness of increasing enzyme concentration diminished. This phenomenon reflected the pH-dependent activity limits of enzymes and was in agreement with Akyüz and Ersus (2021), in which increasing enzyme dosage was effective only when the reaction environment supported the active conformation of the enzyme [24]. In contrast, interactions such as pH–time (X_1_X_4_) and enzyme concentration–time (X_3_X_4_) exhibited smaller response amplitudes, indicating moderate effects on protein content. Although extending the hydrolysis time could enhance protein release, protein content tended to decrease slightly when the reaction time exceeded the optimal duration. This behavior was probably associated with excessive hydrolysis or partial protein denaturation. The interactions between temperature and enzyme concentration (X_2_X_3_) and between temperature and hydrolysis time (X_2_X_4_) also showed increasing trends in protein content within the optimal temperature range, then decreased when temperature exceeded the thermal stability threshold of the enzyme.

Overall, Figure 3 confirms that protein content was strongly influenced by the interactions among factor pairs. Interactions involving pH, particularly X_1_X_2_ and X_1_X_3_, played dominant roles, which agreed with ANOVA and Pareto analyses presented earlier. These findings emphasized the necessity of applying RSM to simultaneously optimize protein extraction conditions instead of optimizing individual parameters independently.

**Figure 4:**
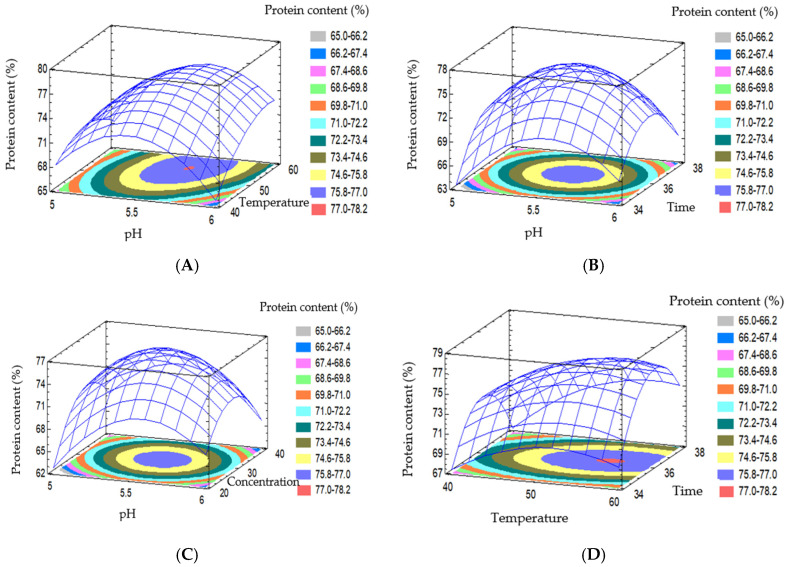

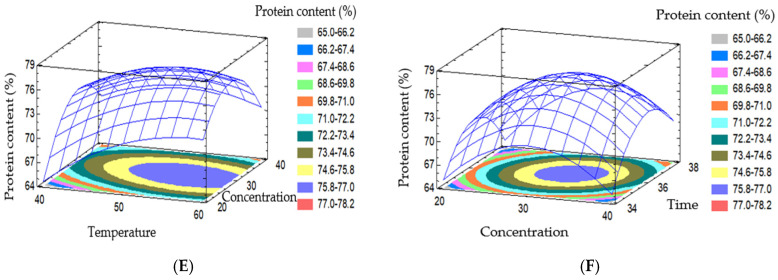
Response surface graph showing the influence of factors and interactions on protein content. (**A**) Enzyme concentration of 30 U/g; time of 36 h. (**B**) Temperature of 50 °C; enzyme concentration of 30 U/g. (**C**) Temperature of 50 °C; time of 36 h. (**D**) pH of 5.5; enzyme concentration of 30 U/g. (**E**) pH of 5.5; time of 36 h. (**F**) pH of 5.5; temperature of 50 °C.

Figure 4 presents the effects of pH, temperature, enzyme concentration, and hydrolysis time, as well as their interactions on protein content. All response surfaces exhibited pronounced curvature, while the contour plots displayed elliptical shapes, indicating the presence of optimal regions and nonlinear relationships between processing parameters and protein content. These results were consistent with the second-order regression model and ANOVA. At an enzyme concentration of 30 U/g and a hydrolysis time of 36 h (Figure 4A), the interaction between pH and temperature showed that the protein content reached its maximum in the pH range of approximately 5.5–6.0, with a temperature of around 50 °C. Deviations in either pH or temperature from this optimal region resulted in decreasing protein content, indicating reduced enzyme activity and potential protein denaturation. When the temperature was maintained at 50 °C and the enzyme concentration at 30 U/g (Figure 4B), the interaction between pH and hydrolysis time demonstrated that protein content increased up to an optimal point, then slightly decreased as the reaction time prolonged. This behavior could be attributed to progressive enzyme deactivation over time or excessive protein hydrolysis into small soluble peptides, leading to a reduction in measurable protein content. A similar phenomenon was reported by Amiri et al. (2024) during enzymatic protein extraction from plant biomass [21].

Figure 4C illustrates the interaction between pH and enzyme concentration under fixed temperature and time conditions. The results indicated that increasing the enzyme concentration enhanced protein content up to an optimal level, beyond which it produced no substantial improvement. This reflects substrate saturation and enzyme–substrate diffusion limitations, aligning with Akyüz and Ersus (2021), who emphasized the nonlinear influence of enzyme concentration and the necessity of its simultaneous optimization with pH [24].

As shown in Figure 4D–F, the interactions between temperature and time, temperature and enzyme concentration, and enzyme concentration and time all exhibited significant effects on protein content, with distinct maxima observed on the response surfaces. The interaction between enzyme concentration and hydrolysis time highlighted the dominant roles of these two factors in disrupting the substrate structure and facilitating protein release.

Overall, the results presented in Figure 4 demonstrated that protein content was strongly influenced by the interactions among processing parameters, with temperature and enzyme concentration playing dominant roles, followed by hydrolysis time, while pH primarily functioned as a modulating factor of the reaction environment. These findings confirmed the suitability of multivariate optimization using RSM and provided a scientific basis for selecting optimal conditions to enhance protein content during the extraction process.

**Figure 5:**
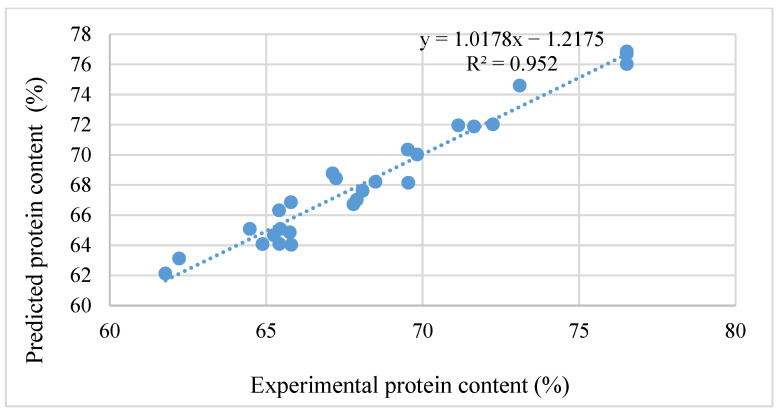
Protein content compatibility between experimental and predicted values.

Figure 5 illustrates the correlation between the experimental protein values and those predicted by the regression model. The regression line followed the equation y = 1.0178x − 1.2175, with a coefficient of determination (R^2^) of 0.952, indicating a high degree of model fitness (95.2%) between experimental and predicted results. This finding confirmed that the response surface model accurately described the relationship between the independent variables (pH, temperature, enzyme concentration, and reaction time) and the obtained protein content. Accordingly, the combined use of response surface plots and the correlation plot supported the conclusion that the developed model was reliable and could be effectively applied to predict and optimize the protein extraction process.

The optimization results presented in Table 3 indicated that the conditions of a pH of 5.56, temperature of 55.2 °C, enzyme concentration of 30.2 U/g, and hydrolysis time of 36.3 h yielded a maximum protein content of 77.1%. These conditions were characteristic of the optimal activity profile of Viscozyme, which exhibits high catalytic performance under mildly acidic conditions at moderate to relatively high temperatures. At pH 5.56, the constituent enzymes of Viscozyme, particularly cellulase and hemicellulase, maintained a stable active conformation, thereby facilitating the disruption of plant cell wall structures and promoting efficient protein release. A temperature of 55.2 °C was considered optimal for Viscozyme catalytic activity, as it enhanced the rate of hydrolytic reactions without inducing protein denaturation or enzyme deactivation. An enzyme concentration of 30.2 U/g ensured sufficient enzyme availability for uniform interaction with the substrate, while a hydrolysis time of 36.3 h was adequate to achieve a balance between reaction kinetics and protein recovery efficiency. Enzymatic extraction typically concentrates the protein in the extract compared to the raw material. For example, treating defatted rice bran with protease (alone) yielded extracts with very high protein concentration up to ~28.5% protein for green (immature) bran and ~23.4% for mature bran versus only ~7–8% protein in non-enzymatic controls [11]. In contrast, multi-enzyme treatments often give different results. In one study, an α-amylase–protease sequence (e.g., starch removal followed by protease) produced extracts with moderate protein (around 14–19%), compared with ~23–28% from protease alone [11]. In barley, a two-step (bi-enzymatic) protocol using starch-hydrolyzing enzymes followed by protease yielded a protein concentrate containing ~49.0% protein [26]. Overall, these optimal conditions reflected the synergistic activity of Viscozyme, including enhanced polysaccharide matrix degradation and liberated free proteins, thus achieving maximum protein recovery efficiency.

### 3.2. Effect of Cellulose Hydrolysis Conditions in VD20 Broken Rice Flour Using Viscozyme on Protein Recovery Efficiency

Protein recovery efficiency was evaluated in parallel with protein content using the same Box–Behnken matrix, and a separate quadratic model was fitted for this response.

The analysis of variance results of the second-order regression model for optimizing protein recovery efficiency demonstrated that the model adequately described the relationship between the technological factors and the studied response (Table 4). The ANOVA results showed that the quadratic regression model adequately described protein recovery efficiency. Among the linear terms, only temperature was statistically significant, with F = 134.37 and *p* = 0.0074, indicating its major role in governing enzyme activity and disruption of the substrate matrix during hydrolysis. In contrast, pH, enzyme concentration, and hydrolysis time were not significant as individual linear terms at the 95% confidence level. However, several interaction terms, namely, pH–temperature, pH–enzyme concentration, temperature–enzyme concentration, and enzyme concentration–time, were significant, indicating that recovery efficiency depended on the combined reaction conditions rather than on isolated factors alone [27]. All quadratic terms were highly significant, confirming the nonlinear nature of the response and indicating that recovery efficiency increased to a maximum within a limited operating region before declining beyond the optimum. Such behavior is typical of enzyme-assisted hydrolysis systems, in which activity and stability are maintained only within a restricted range of conditions [28]. The lack-of-fit value of 0.0763 (>0.05) indicated no significant difference between the regression model and the experimental results, thereby confirming that the model was suitable for prediction and optimization within the studied range.

To describe the relationship between cellulose hydrolysis conditions and protein recovery efficiency, a regression equation was established:

(2) Protein recovery efficiency (%)  = −3130.63 + 368.88X_1_ + 3.45083X_2_ + 3.8045X_3_ + 112.976X_4_ − 34.7783X_1_^2^ + 0.4755X_1_X_2_ − 0.3385X_1_X_3_ + 0.0275X_1_X_4_ − 0.0433708X_2_^2^ − 0.021525X_2_X_3_ − 0.024875X_2_X_4_ − 0.0676958X_3_^2^ + 0.089625X_3_X_4_ − 1.59427X_4_^2^

where X_1_ represents the pH (5.0–6.0);

X_2_ represents the temperature (40–60 °C);

X_3_ represents the cellulase enzyme concentration (20–40 U/g);

X_4_ represents the hydrolysis time (34–38 h).

A Pareto chart was also constructed to rank the relative importance of the model terms affecting protein recovery efficiency.

**Figure 6:**
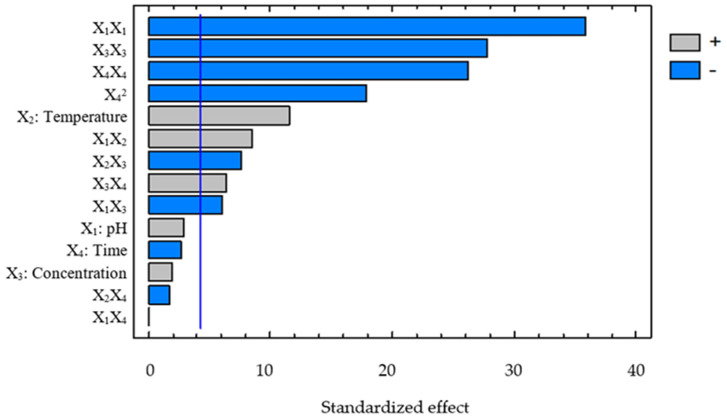
The effects of variables on protein recovery efficiency during cellulose hydrolysis via Viscozyme.

Figure 6 ranks the effects of the process variables and their interactions on protein recovery efficiency during Viscozyme-assisted hydrolysis. The quadratic terms were dominant, indicating the presence of a clear optimum region rather than a simple linear increase in response. Similar trends have been reported in recent studies on enzymatic extraction of plant proteins, where quadratic regression models effectively described the variation of yield with processing conditions [29]. Among the linear effects, temperature remained the most influential factor because it directly affected catalytic activity and structural stability of the enzyme, thereby governing matrix disruption and protein release. The significant interaction terms further indicated that recovery efficiency depended on the combination of processing conditions, especially pH with temperature, pH with enzyme concentration, temperature with enzyme concentration, and enzyme concentration with time [27]. In contrast, the interactions between pH and time and between temperature and time had relatively minor effects within the investigated range.

Overall, the Pareto analysis confirmed that protein recovery efficiency during Viscozyme-assisted hydrolysis resulted from the combined effects of multiple variables and their complex interactions. The inclusion of linear, quadratic, and interaction terms was essential for developing a reliable optimization model that could contribute to improved utilization of protein from broken rice as a promising by-product in agro-food processing.

**Figure 7:**
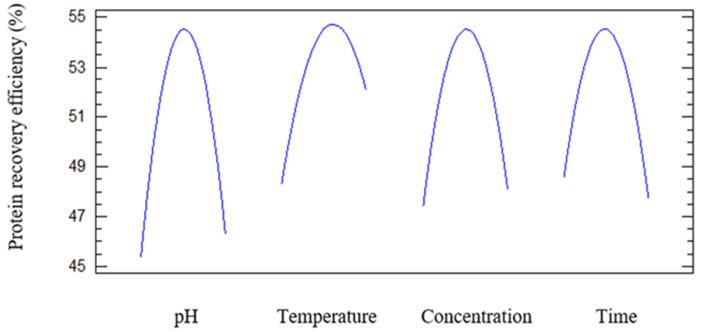
Effects of pH, temperature, concentration, and time on protein recovery efficiency.

Figure 7 shows downward-opening parabolic curves for all four variables, indicating that protein recovery efficiency increased to an optimum and then decreased once the operating conditions exceeded the favorable range. This pattern was consistent with the significant quadratic terms obtained from the regression model. Under unfavorable pH conditions, enzyme denaturation occurred, leading to a decrease in protein recovery efficiency. This trend was consistent with the findings of Zhang et al. (2018), who reported that the optimal pH for enzyme-assisted extraction of plant proteins generally ranged from 5.0 to 6.5 with Viscozyme [16]. Regarding temperature, protein recovery efficiency increased as the temperature rose to an optimal value and then decreased markedly as the temperature continued to increase. This pattern reflected the balance between enhanced enzymatic reaction rates and the risk of enzyme inactivation at elevated temperatures.

Recovery efficiency increased initially with increasing enzyme concentration because more catalytic sites were available, then slowly declined due to substrate saturation and diffusion limitations [24]. Similar optimum-type behavior was observed for pH, temperature, and hydrolysis time, reflecting the balance between enhanced hydrolysis and losses associated with enzyme deactivation or excessive protein degradation [21].

**Figure 8:**
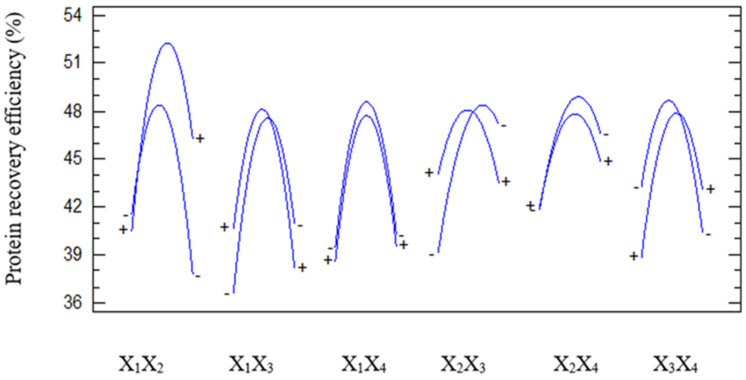
Graph showing the effect of each pair of factors on protein recovery efficiency.

Figure 8 illustrates the pairwise interaction effects among the processing variables on protein recovery efficiency. The nonlinear and non-parallel response surfaces indicated that the factors acted simultaneously during hydrolysis, aligning with the significant interaction terms in the regression model. The pH–temperature combination produced the clearest optimum region, with maximum recovery observed under slightly acidic to near-neutral conditions at moderate temperature. Other interaction surfaces showed the same general pattern, namely, improvement up to an optimum followed by stabilization or decline when one factor exceeded the favorable range. These results reflected the combined dependence of enzyme conformation, catalytic activity, and protein solubility on the reaction environment [16].

**Figure 9:**
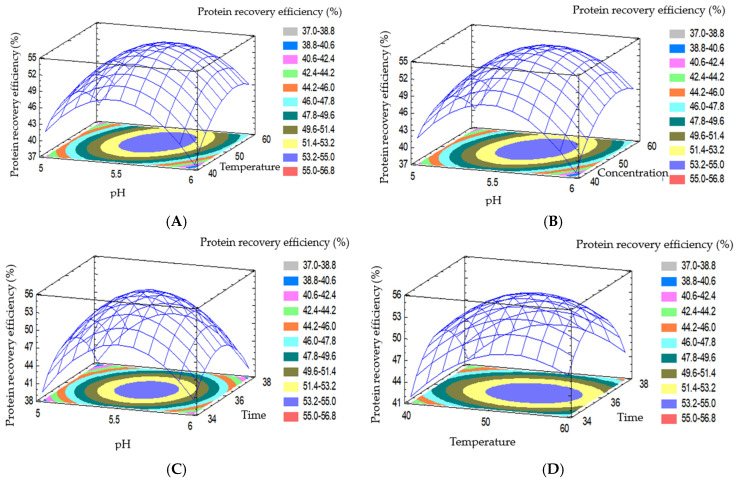

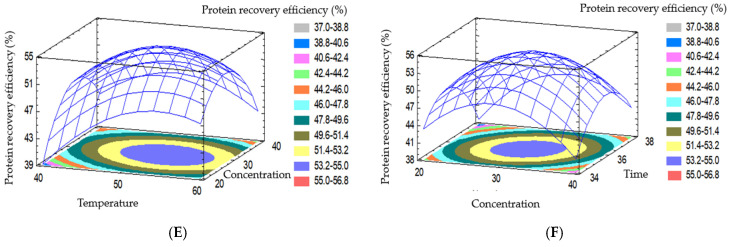
The influence of factors and interactions on protein recovery efficiency. (**A**) Enzyme concentration of 30 U/g; time of 36 h. (**B**) Temperature of 50 °C; time of 36 h. (**C**) Temperature of 50 °C; enzyme concentration of 30 U/g. (**D**) pH of 5.5; enzyme concentration of 30 U/g. (**E**) pH of 5.5; time of 36 h. (**F**) pH of 5.5; temperature of 50 °C.

Figure 9 presents the corresponding response surface and contour plots for protein recovery efficiency. The pronounced curvature of the surfaces and the predominantly elliptical contours confirmed the existence of distinct optimal regions and supported the use of the second-order model. For example, at a fixed enzyme concentration and hydrolysis time, the pH–temperature surface showed maximum recovery near pH 5.5–6.0 and around 50 °C. The temperature–time and enzyme concentration–time surfaces also displayed clear maxima, indicating that prolonged hydrolysis was beneficial only within a suitable temperature and dosage range. Beyond that region, the gain diminished because of enzyme deactivation, substrate limitation, or excessive hydrolysis of the extracted protein. Overall, the interaction plots confirmed that protein recovery efficiency was governed by the combined effects of the processing variables, with temperature and hydrolysis time exerting the strongest practical influence and pH mainly regulating the reaction environment.

**Figure 10:**
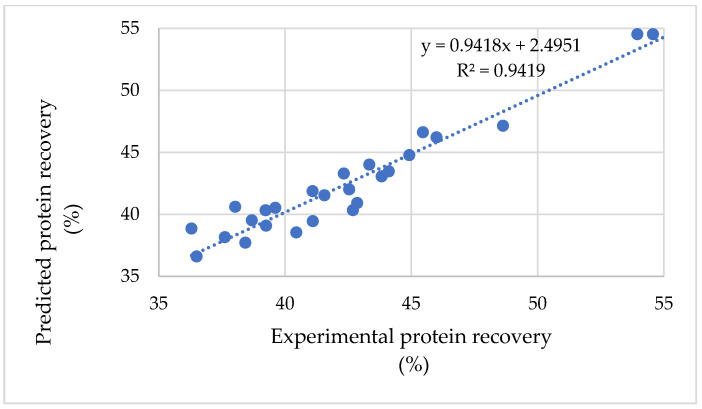
The compatibility of protein recovery efficiency between experimental and predicted results.

Figure 10 illustrates the linear relationship between the experimental protein recovery efficiency and the values predicted by the quadratic regression model. The obtained regression equation was y = 0.9418x + 2.5951, with a coefficient of determination R^2^ = 0.9419, indicating a strong correlation between predicted and experimental values. The slope being slightly lower than 1 suggested that the model tended to underestimate responses at higher values, while the positive intercept indicated a slight overestimation at lower values. However, these deviations were minor, demonstrating that the model exhibited high reliability and could be used for predicting protein recovery efficiency. Therefore, the quadratic regression model applied in this study was considered appropriate for describing and predicting protein recovery efficiency, thereby supporting the identification of optimal processing conditions for Viscozyme-assisted enzymatic hydrolysis. The efficiency of protein recovery also varies depending on the enzymes used and processing conditions. In the rice bran example above, protease-assisted extraction recovered only ~8.5% of the total protein from green bran, compared with ~3% for the non-enzymatic treatment [11]. In contrast, optimization using design-of-experiments can greatly increase efficiency; for instance, a Box–Behnken RSM study of wheat bran extraction achieved about 53.9% protein recovery [30]. Multi-enzyme schemes have been shown to boost recovery; a tri-enzymatic barley extraction (starch hydrolases, protease and glucanase) recovered about 78.3% of the total barley protein [26]. Based on the predictive model, the optimal conditions for cellulose hydrolysis leading to maximum protein recovery efficiency were determined. As shown in Table 5, the model predicted a maximum protein recovery efficiency of 54.8% at a pH of 5.53, temperature of 52.4 °C, cellulase enzyme concentration of 29.7 U/g, and hydrolysis time of 35.9 h.

### 3.3. Optimization of Two Cellulose Hydrolysis Surfaces for Protein Content and Protein Recovery Efficiency

The optimization results of the cellulose hydrolysis process under optimal conditions for each specific objective were obtained by optimizing the individual response surfaces of protein content and protein recovery efficiency, as well as by simultaneously optimizing both responses. The effects of pH, temperature, enzyme concentration, and hydrolysis time were evaluated, and the optimization outcomes are illustrated in Figure 6. Optimization of the cellulose hydrolysis process was conducted following two approaches: (i) individual optimization for each response (protein content and protein recovery efficiency) and (ii) simultaneous optimization of both responses using RSM. Table 6 summarizes the corresponding optimal conditions of pH, temperature, enzyme concentration, and hydrolysis time identified for each optimization strategy.

The optimal conditions obtained from single-response optimization for maximizing protein content (%) were identified as a pH of 5.56, temperature of 55.2 °C, enzyme concentration of 30.2 U/g, and hydrolysis time of 36.2 h, under which the highest protein content of 77.1% was achieved.

When protein recovery efficiency was optimized individually, the reaction conditions shifted to a pH of 5.53, temperature of 52.4 °C, enzyme concentration of 29.7 U/g, and hydrolysis time of 35.9 h, resulting in a protein recovery efficiency of 54.8%. The differences between these two sets of optimal conditions indicated that each response variable required a distinct catalytic environment. Consequently, single-objective optimization could lead to discrepancies in the overall process performance. The simultaneous optimal conditions for both responses were determined as a pH of 5.53, temperature of 52.6 °C, enzyme concentration of 29.8 U/g, and hydrolysis time of 35.9 h. Under these combined conditions, a protein content of 76.9% and a protein recovery efficiency of 54.8% were obtained, representing only slight reductions compared with the respective maximum values achieved through individual optimization. Overall, the optimization analysis clearly revealed substantial differences between the optimal conditions derived from single-response optimization and those obtained through simultaneous optimization.

**Figure 11:**
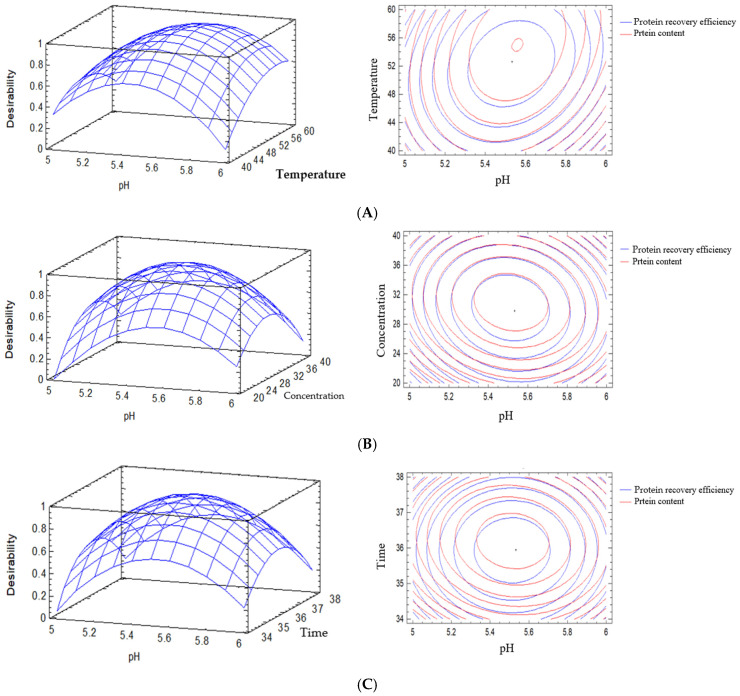

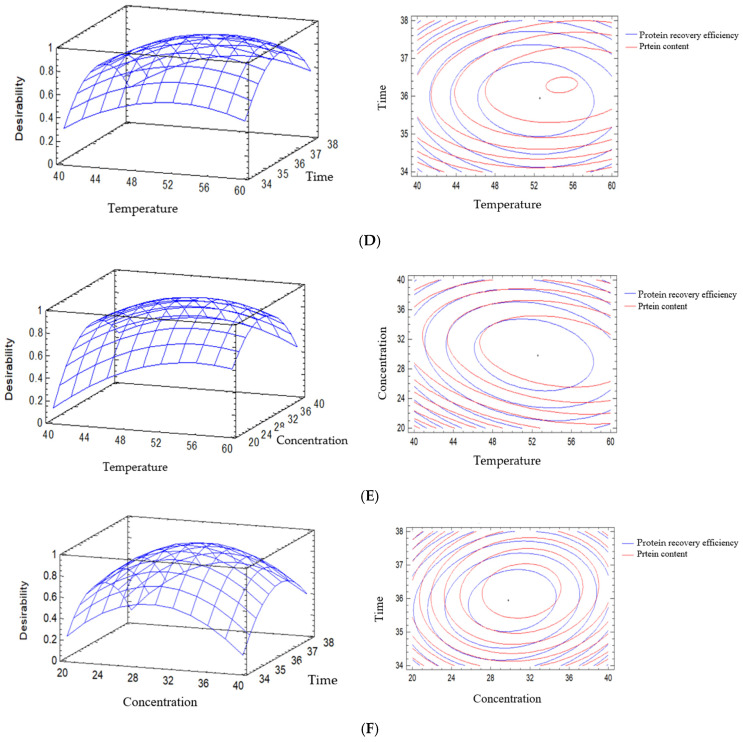
The influence of factors and interactions on protein content and protein recovery efficiency. (**A**) Enzyme concentration of 30 U/g; time of 36 h. (**B**) Temperature of 50 °C; time of 36 h. (**C**) Temperature of 50 °C; enzyme concentration of 30 U/g. (**D**) pH of 5.5; enzyme concentration of 30 U/g. (**E**) pH of 5.5; time of 36 h. (**F**) pH of 5.5; temperature of 50 °C.

Figure 11 presents the simultaneous effects of the main processing variables, including pH, temperature, enzyme concentration, and hydrolysis time, on protein content (%) and protein recovery efficiency. All response surfaces exhibited pronounced curvature, and the contour plots were elliptical or oval-shaped with concentrated distributions, confirming the nonlinear relationships and the existence of optimal regions for the investigated variables. These results were consistent with the second-order regression model and the ANOVA analyses presented in Table 2 and Table 4, in which both quadratic and interaction terms were statistically significant.

At a fixed enzyme concentration of 30 U/g and a hydrolysis time of 36 h (Figure 11A), the interaction between pH and temperature showed that protein content reached its maximum value within a pH range of 5.5–6.0 at 50 °C. Deviations in either pH or temperature from this optimal region resulted in a gradual decline in protein content, reflecting the sensitivity of enzymes to pH and temperature conditions. When the temperature was fixed at 50 °C and the hydrolysis time at 36 h (Figure 11B), the interaction between pH and enzyme concentration exhibited a nonlinear relationship, whereby increasing the enzyme concentration enhanced protein content only within the optimal pH range. This observation was similar to that of Akyüz and Ersus (2021), who reported that enzyme concentration improved protein extraction efficiency only within the permissible pH activity window [24].

For protein recovery efficiency, the interaction between pH and temperature (Figure 11A) also produced concentrated contour lines with a maximum region similar to that observed for protein content, indicating that the optimal conditions for protein content closely corresponded to those for protein recovery efficiency. The pH–enzyme concentration interaction for protein recovery efficiency showed that efficiency increased when both factors were adjusted within their optimal ranges. However, outside these ranges, protein recovery efficiency declined more rapidly than protein content (Figure 11B). When examining the interactions between temperature–time and enzyme concentration–time (Figure 11C,D), both protein content and protein recovery efficiency increased with increasing hydrolysis time within the optimal range. Additionally, the pH–time (Figure 11E) and enzyme concentration–temperature (Figure 11F) interactions demonstrated the combined dependence of both responses on these factor pairs. Each interaction generated a defined optimal region on the response surfaces, emphasizing the necessity of simultaneous multivariable optimization rather than independent adjustment of individual factors. Overall, Figure 11 shows that both protein content and protein recovery efficiency were strongly influenced by the combined effects of pH, temperature, enzyme concentration, and hydrolysis time. These findings confirmed that the effects of processing variables on protein extraction performance were inherently nonlinear, and the integrated optimal conditions existed for both responses [16,24]. The application of RSM-based optimization not only improved protein content but also maximized protein recovery efficiency while maintaining reaction conditions within a safe operational range for enzyme activity. Accordingly, the optimal conditions for protein extraction from VD20 broken rice were determined as a pH of 5.55, temperature of 53.1 °C, Viscozyme concentration of 29.4 U/g, and hydrolysis time of 35.8 h, resulting in protein recovery efficiency of 24.4% and protein content of 76.8%. The optimal protein content of 76 to 77% and recovery efficiency of 54.8% obtained in this study were slightly higher than those reported in recent studies on rice protein extraction. For example, enzymatic extraction from rice materials typically yielded protein contents in the range of 50 to 70%, depending on processing conditions [31,32]. A recent study reported a protein content of approximately 70% with a recovery efficiency of up to 73.96% under optimized enzymatic conditions [33]. The protein content obtained in the present work was slightly higher, indicating an effective purification process. The recovery efficiency of 54.8% fell within the commonly reported range of enzyme-assisted extraction [32]. Although some optimized processes have achieved higher recovery efficiency, the results of this study demonstrated a suitable balance between protein purity and recovery efficiency. The differences among studies could be attributed to variations in raw materials, enzyme composition, and processing parameters.

## 4. Conclusions

This study demonstrated that Viscozyme-assisted hydrolysis is an effective strategy for enhancing protein recovery from VD20 broken rice flour. The main scientific contribution lies in identifying nonlinear interactions among process variables and establishing optimized conditions through RSM, including a multi-response approach that balanced protein content and recovery efficiency. The optimized process achieved high protein content and improved recovery efficiency, confirming the effectiveness of simultaneous optimization for practical process design. These findings highlight the potential of this approach for converting rice by-products into protein-rich ingredients with industrial relevance. Future work should focus on process scale-up, improving cost efficiency, and evaluating the functional properties of the recovered protein for food and feed applications.

## Figures and Tables

**Table 1:** Factors and levels of investigation in the experiment to optimize the hydrolysis of cellulose in VD20 rice flour using the Box–Behnken model.

Symbol	Factor	Unit	Level		
			−1	0	1
X_1_	pH		5.0	5.5	6.0
X_2_	Temperature	°C	40	50	60
X_3_	Enzyme concentration	U/g	20	30	40
X_4_	Time	h	34	36	38

**Table 2:** ANOVA analysis results for protein content values.

Source	Sum of Squares	df	Mean Square	F-Value	*p*-Value
X_1_: pH	2.63203	1	2.63203	13.33	0.0675
X_2_: Temperature	41.7014	1	41.7014	211.22	0.0047
X_3_: Enzyme concentration	13.889	1	13.889	70.35	0.0139
X_4_: Time	13.188	1	13.188	66.80	0.0146
X_1_^2^	168.875	1	168.875	855.35	0.0012
X_1_X_2_	12.1801	1	12.1801	61.69	0.0158
X_1_X_3_	7.2361	1	7.2361	36.65	0.0262
X_1_X_4_	2.4025	1	2.4025	12.17	0.0733
X_2_^2^	23.1481	1	23.1481	117.25	0.0084
X_2_X_3_	10.7912	1	10.7912	54.66	0.0178
X_2_X_4_	4.1209	1	4.1209	20.87	0.0447
X_3_^2^	148.31	1	148.31	751.19	0.0013
X_3_X_4_	4.7961	1	4.7961	24.29	0.0388
X_4_^2^	142.945	1	142.945	724.02	0.0014
Lack-of-fit	34.7578	10	3.47578	17.60	0.0549
Pure error	0.394867	2	0.197433		
Total (corr.)	433.252	26			

**Table 3:** Results of protein content optimization (%).

Factors	Optimal Value
pH	5.56
Temperature (°C)	55.2
Enzyme concentration (U/g)	30.2
Hydrolysis time (h)	36.3
Protein content (%)	77.1

**Table 4:** Results of ANOVA analysis of the quadratic regression model for optimizing protein recovery efficiency.

Source	Sum of Squares	df	Mean Square	F-Value	*p*-Value
X_1_: pH	2.58541	1	2.58541	8.21	0.1032
X_2_: Temperature	42.3001	1	42.3001	134.37	0.0074
X_3_: Concentration	1.17188	1	1.17188	3.72	0.1935
X_4_: Time	2.22741	1	2.22741	7.08	0.1170
X_1_^2^	403.177	1	403.177	1280.74	0.0008
X_1_X_2_	22.61	1	22.61	71.82	0.0136
X_1_X_3_	11.4582	1	11.4582	36.40	0.0264
X_1_X_4_	0.003025	1	0.003025	0.01	0.9309
X_2_^2^	100.322	1	100.322	318.68	0.0031
X_2_X_3_	18.533	1	18.533	58.87	0.0166
X_2_X_4_	0.990025	1	0.990025	3.14	0.2182
X_3_^2^	244.412	1	244.412	776.40	0.0013
X_3_X_4_	12.8522	1	12.8522	40.83	0.0236
X_4_^2^	216.892	1	216.892	688.98	0.0014
Lack-of-fit	39.3588	10	3.93588	12.50	0.0763
Pure error	0.6296	2	0.3148		
Total (corr.)	688.184	26			

**Table 5:** Optimization of protein recovery efficiency.

Factors	Optimal Value
pH	5.53
Temperature (°C)	52.4
Enzyme concentration (U/g)	29.7
Hydrolysis time (h)	35.9
Protein recovery efficiency (%)	54.8

**Table 6:** Effect of the process when optimizing two cellulose hydrolysis surfaces on protein content and protein recovery efficiency.

Factors	Optimization of Each Surface		Optimization of Two Surfaces
	Protein Content (%)	Recovery Efficiency (%)	
pH	5.56	5.53	5.53
Temperature (°C)	55.2	52.4	52.6
Enzyme concentration (U/g)	30.2	29.7	29.8
Hydrolysis time (hour)	36.2	35.9	35.9
Optimization results for each surface	77.1	54.8	
Optimization results for two surfaces	76.9	54.8	

## Data Availability

The original contributions presented in this study are included in the article. Further inquiries can be directed to the corresponding author.
